# N-3 Polyunsaturated Fatty Acids Ameliorate Neurobehavioral Outcomes Post-Mild Traumatic Brain Injury in the *Fat-1* Mouse Model

**DOI:** 10.3390/nu13114092

**Published:** 2021-11-15

**Authors:** Jessica-Dominique Lecques, Brynna J. K. Kerr, Lyn M. Hillyer, Jing X. Kang, Lindsay E. Robinson, David W. L. Ma

**Affiliations:** 1 Department of Human Health and Nutritional Sciences, University of Guelph, Guelph, ON N1G 2W1, Canada; jlecqu01@guelphhumber.ca (J.-D.L.); brynna@uoguelph.ca (B.J.K.K.); lhillyer@uoguelph.ca (L.M.H.); lrobinson@uoguelph.ca (L.E.R.); 2 Massachusetts General Hospital and Harvard Medical School, Boston, MA 02114, USA; kang.jing@mgh.harvard.edu

**Keywords:** concussion, mild traumatic brain injury, TBI, n-3 PUFA, neurological sensitivity score

## Abstract

Concussions and mild traumatic brain injury (m-TBI) have been identified as a consequential public health concern because of their potential to cause considerable impairments in physical, cognitive, behavioral, and social functions. Given their prominent structural and functional roles in the brain, n-3 polyunsaturated fatty acids (PUFA) have been identified as a potentially viable prophylactic agent that may ameliorate the deleterious effects of m-TBI on brain function. The purpose of the present pilot study was to investigate the effect of n-3 PUFA on neurologic function using a weight drop injury (WDI) model. *Fat-1* mice, capable of synthesizing n-3 PUFA endogenously from n-6 PUFA, and their wild-type (WT) counterparts, were subjected to a mild low-impact WDI on the closed cranium, and recovery was evaluated using the neurological severity score (NSS) to assess the motor and neurobehavioral outcomes. In comparison to the WT mice, the *fat-1* mice had a significantly (*p* ≤ 0.05) lower NSS at all time points post-WDI, and significantly greater neurological restoration measured as the time to first movement. Overall, these findings demonstrate the protective effect of n-3 PUFA against mild brain injury.

## 1. Introduction

Concussions have been identified as a consequential public health concern because of their potential to cause considerable impairments in physical, cognitive, behavioral, and social functions [1]. Traumatic brain injury (TBI) is induced by acceleration/deceleration forces, blunt and/or penetrating trauma that interrupt normal brain function [1,2]. In the United States (USA), upwards of 3.5 million TBIs are reported annually, accounting for approximately 52,000 deaths, and greater than 300,000 hospitalizations [3]. It is estimated that concussions constitute approximately 80 to 95% of all cases of TBI that receive medical attention, and account for 44% of the 60 billion USD annual healthcare costs of TBI in the USA [2,3]. However, because numerous patients with concussion do not seek professional treatment, the prevalence of all concussions among adults is presumed to be greater than reported [4]. As such, it is evident that concussions are a health epidemic that impose a substantial economic burden. 

There is recognition within the medical literature that a concussion is typically a less severe form of TBI [5]. However, the use of multiple terms (closed-head injury, subconcussive, concussion, and mild traumatic brain injury (mTBI)) describing the spectrum of severities underscores a range of injury types [5]. The Centers for Disease Control and Prevention, for instance, previously provided a comprehensive and collective definition for "concussion" and "mild traumatic brain injury", using the two terms interchangeably: [6]. mTBI results in altered brain function and a constellation of symptoms that may impair physical, cognitive, emotional, and other behavioral functions [7]. 

While a definitive intervention to treat the effects of TBI remains elusive, dietary supplements may be a promising avenue to protect against and mitigate brain injury from concussions. Emerging research suggest that n-3 polyunsaturated fatty acids (PUFA), including docosahexaenoic acid (DHA, 22:6n3), and eicosapentaenoic acid (EPA, 20:5n3), may be useful in protecting the brain from acute TBI [7,8,9,10]. This is biologically plausible given that up to 97% of all n-3 PUFA in the brain are comprised of DHA [8,10]. Specifically, EPA and DHA have been shown to mitigate the adverse mechanisms of the secondary phase of TBI by diminishing excitotoxicity, microglial activation, axonal damage, apoptosis, and hypermetabolism [10]. 

While there is emerging evidence supporting the prophylactic effects of n-3 PUFA on mTBI, additional mechanistic studies are needed to show causal relationships. A potential model for such work is the *fat-1* mouse model, which is capable of the de novo synthesis of n-3 PUFA. In the present study, *fat-1* mice were subjected to a weight drop injury (WDI) to investigate the effect of n-3 PUFA on mTBI prognosis [11,12]. To quantify the severity of injury and functional recovery, a post-traumatic assessment of the motor and neurobehavioral impairment was assessed utilizing a neurological severity score (NSS). Further, an immunohistochemical analysis was performed to determine the quantity of cerebral microhemorrhages post-WDI.

## 2. Materials and Methods 

### 2.1. Animals and Experimental Design

Male *fat-1* mice were mated with C57/Bl female mice (Charles River) to generate *fat-1* and WT offspring. The mice were weaned at 3 weeks of age and maintained on a 10% *w*/*w* safflower diet (Research Diets, New Brunswick, NJ, USA) and distilled water ad libitum. The mice were group-housed (3–4 mice per cage) and the ambient temperature was controlled at 22–24 °C, with a standard 12 h light/dark cycle; all testing was conducted during the light phase of the cycle. In this pilot study, both males and females were used and pooled together. At 7 to 8 weeks of maturity, WT (3 male and 3 female) and *fat-1* (2 male and 4 female) offspring were subjected to a WDI and then assessed for motor and neurobehavioral function over 168 h (1 week), and then terminated for tissue collection. The time to first movement was assessed post-WDI as an initial measure of the neurological restoration (7 male and 5 female WT; 6 female and 6 male *fat-1).* A second set of mice received a WDI and were terminated at 1 h (1 male and 4 female WT; 3 male and 2 female *fat-1*), and 168 h (4 male and 2 female WT; 3 male and 4 female *fat-1*) to assess cerebral microhemorrhages. The NSS scores were also assessed for sham mice not receiving a WDI at 1 h (1 male and 1 female *fat-1*; 1 male WT), and 24 h (1 male WT; 1 male *fat-1*). The sham WT and *fat-1* mice were pooled together for analysis as they were not different. A subset of mice was used for brain fatty acid analysis (3 female WT; 3 female *fat-1)*.

### 2.2. Ethics

All animal care, handling, and experimental procedures were conducted in compliance and with the approval of the Animal Care and Use Committee of the University of Guelph (#4207).

### 2.3. Administration of Traumatic Brain Injury and Evaluating Time to First Movement

mTBI was induced in mice using a WDI model, as validated by Flierl et al. (2009) [12]. Mice were preoxygenated at 2 L/min O_2_ in a nose cone for approximately 1 min, and subsequently anesthetized with 4% Baxter isoflurane (Cat #19476, CMDV, St. Hyacinthe, QC, Canada) vaporized in oxygen. Once the surgical plane was attained (approximately 40 s to 2 min), the concentration of isoflurane was diminished to a maintenance dosage of 1.5% until the mice’s pedal reflexes were absent. Exposure to 1.5% isoflurane, vaporized in 2 L/min O_2_ via the nose cone, was terminated immediately preceding the delivery of the WDI. A single concussive impact was performed by suspending the unresponsive mouse chest-down onto a stage consisting of a slit piece of aluminum foil, 10 cm above a foam cushion, held in position by a Plexiglas structure. The mouse was positioned onto the aluminum platform such that the 100 g steel weight (1.3 cm × 28 cm) would fall directly onto the dorsal surface of the scalp midline between the bregma and lambda [11]. The mTBI was then induced in the unrestrained mouse by performing a swift upward pull of the weight attached by a nylon fly fishing line. The weight was subsequently released vertically from a height of 163 cm through a PVC guide tube (20 mm × 163 cm), delivering a clinically relevant force of impact onto the mouse’s closed cranium [11]. 

Immediately post-WDI, the mouse was transferred to a holding cage. Then, the time it took for the mouse to perform slight movements was measured. The time to first movement was assessed as an indication of neurological restoration, similar to the righting reflex measured in Kane et al. [11]. 

### 2.4. Assessment of Motor and Neurobehavioral Function 

To assess the functional recovery and the severity of the mTBI imparted by the weight drop apparatus, mice were evaluated by utilizing a set of clinical criteria. The NSS is comprised of a battery of ten individual clinical tasks that evaluate motor and neurobehavioral performance developed by Flierl et al. [12]. Each of the 10 tests was assigned a score of either 0 or 1, contingent on successful task completion or failure, respectively. Thus, a maximal NSS of 10 points indicates an inability to complete all tasks and is, therefore, representative of severe neurological dysfunction [12,13]. By comparison, a score of zero reflects healthy neurological function. The NSS evaluations were conducted in *fat-1* and WT mice at the following time points post-WDI: 1, 4, 24, 48, 72, and 168 h. 

### 2.5. Brain Fatty Acid Analysis

Fatty acid composition determination by gas liquid chromatography has been previously described [14]. Briefly, total lipids were extracted from the brain by the Folch method [14]. The right hemisphere of the brain was homogenized in 1 mL 0.1M potassium chloride (KCl) (EMD Millipore Sigma Cat #PX1405-1, Burlington, VT, USA). A total of 100 μL of homogenate was mixed with 900 μL 0.1M KCl, and 4 mL of chloroform:methanol (2:1), and left to incubate at 4 °C overnight. The next day, samples were centrifuged at 357× *g* for 10 min. The bottom layer was collected and dried down under nitrogen gas. Samples were saponified by adding 2 mL 0.5M potassium hydroxide (KOH) (Cat #P250-500, Fisher Scientific, Pittsburgh, PA, USA) in methanol and heating at 100 °C for 1 h. Methylation was performed by adding 2 mL of hexane and 2 mL of 14% boron trifluoride-methanol (BF3-MeOH) (EMD Millipore Sigma Cat #B1252, Burlington, VT, USA) to the samples and incubating them at 100 °C for 1 h. Following methylation, 2 mL of double-distilled H_2_O was added to the samples and the solution was immediately vortexed for 30 s to halt methylation. Samples were centrifuged for 10 min at 357× *g*, and the hexane layer (top) was collected and dried down under nitrogen before reconstitution in 200 µL of hexane. Fatty acid methyl esters were quantified on an Agilent 6890 gas chromatograph, equipped with flame ionization detection, and separated on a DB-FFAP fused-silica capillary column (15 m, 0.1 m film thickness, 0.1 mm i.d.; Agilent Cat #127-32H2, Santa Clara, CA, USA). Samples were injected in 200:1 split mode. The injector and detector ports were set at 250 °C. Fatty acid methyl esters were eluted using a temperature program set initially at 150 °C and held for 0.25 min, increased at 35 °C/min and held at 170 °C for 3 min, increased at 9 °C/min to 225 °C, and finally, increased 80 °C/min to 245 °C and held for 2.2 min. The run time per sample was 12 min. The carrier gas was hydrogen, set to a 30 mL/min constant flow rate. Peaks were identified by the retention times of fatty acid methyl ester standards (Nu-Chek-Prep, Elysian, MN, USA) using EZChrom Elite version 3.2.1 software. The fatty acid results were calculated as percent composition.

### 2.6. Prussian Blue and Nuclear Fast Red Staining for Detection of Cerebral Microhemorrhages

Cerebral microhemorrhages were assessed at 1 and 168 h. In preparation for Prussian blue and nuclear fast red staining, the brain hemispheres were fixed in 4% formaldehyde and processed overnight in a tissue processor. Brain tissues were then embedded in a paraffin block and 5 μm brain sections were cut through the posteroanterior plane on a Leica RM2235 microtome. Free-floating tissue sections were mounted onto ColorFrost Plus microscopic slides (Cat #9951APLUS-006, Fisher Scientific, Pittsburgh, PA, USA). Sections were subsequently deparaffinized and hydrated using distilled water prior to being immersed in a solution of equal parts 20% hydrochloric acid (Cat #A144-500, Fisher Chemical), and 10% potassium hexacyanoferrate(II) trihydrate (Cat #P3289-5G, Sigma-Aldrich Canada Co., Oakville, ON, Canada) for 20 min. Sections were then washed in 3 changes of distilled water and subsequently counterstained with nuclear fast red (Cat #R5463200-500A, RICCA Chemical Company, Arlington, TX, USA) for 5 min before being rinsed in 2 changes of distilled water. Sections were processed through a series of baths in the following order: dehydration in 95% alcohol (Cat #HC5001GAL, Fisherbrand™ HistoPrep™), Pittsburgh, PA, USA; dehydration in 100% alcohol, 2 changes; and 2 changes of xylene (Cat #HC7001GAL, Fisherbrand™ HistoPrep™), 3 min each. After staining, sections were mounted with xylene glue and coverslipped (Cat #12-548-5P, Fisherfinest Premium Cover Glass, Pittsburgh, PA, USA). Hemorrhage profiles were counted by 1 independent observer using a Nikon Eclipse TS100 microscope at 20× the original magnification. For each brain section, the average number of Prussian blue positive clusters were calculated, as described by Fisher et al. [15]. Clusters were defined as regions containing ≥2 iron granules within proximity to one another.

### 2.7. Statistical Analysis

SAS version 9.1 (SAS Institute, Cary, NC, USA) was used for all statistical analyses. Data are presented as mean ± standard error of the mean (SEM). A repeated measures analysis was applied to the total NSS to detect the differences between the WT and the *fat-1* over the 168 h evaluation period. A two-tailed *t*-test was conducted to detect differences in time to first movement following a WDI between WT and *fat-1* mice. Student’s *t*-tests were used to identify differences in the brain fatty acid composition and microhemorrhages in WT and *fat-1* brains. A *p*-value of *p* ≤ 0.05 was considered statistically significant. 

## 3. Results

### 3.1. Time to First Movement

**Figure 1:**
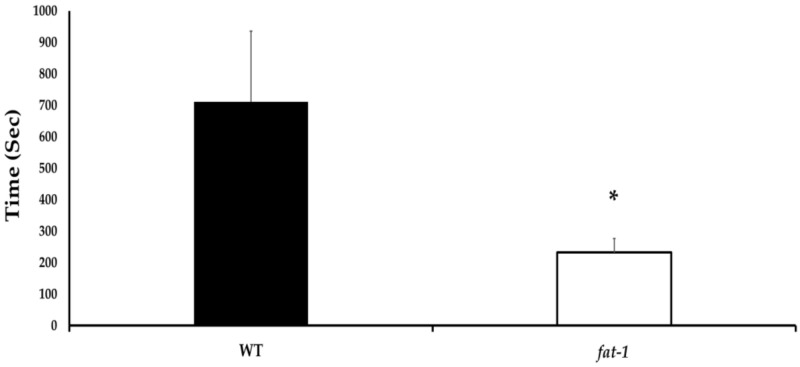
Time to first movement following a WDI, wherein a lower time is correlated with greater neurological restoration (7 male and 5 female WT; 6 male and 6 female *fat-1).* Bars represent mean ± SEM. * denotes a significant difference (*p* ≤ 0.05) by two-tailed *t*-test.

*Fat-1* mice showed significantly faster times to first movement following the WDI (*p* ≤ 0.05). Specifically, it took the WT mice approximately 200% longer to display voluntary movement following the WDI (Figure 1). 

### 3.2. Neurological Severity Score (NSS)

**Figure 2:**
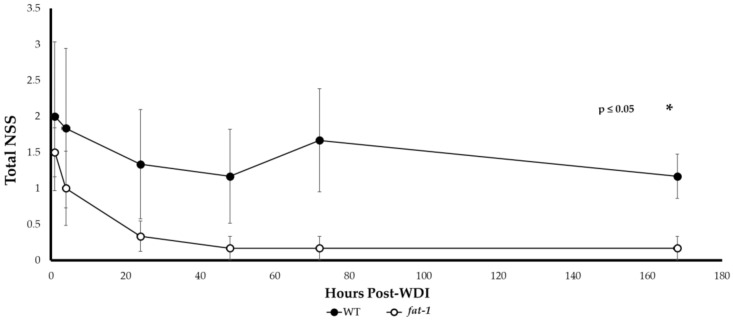
Assessment of recovery by NSS over 168 h -post-WDI. A repeated measures analysis reveals significantly lower NSS (*: *p* ≤ 0.05) for *fat-1* (2 male and 4 female *fat-1*) relative to WT (3 male and 3 female WT) mice across all time points post-WDI. Additionally, a significant difference was also observed at the 168 h time point by two-tailed *t*-test.

Use of the NSS has been previously validated and deemed a highly reliable prognostic tool. Performances on the NSS tasks have been found to be consistent with recovery, as determined by the functional and magnetic resonance imaging (MRI) measures [11,12]. *Fat-1* mice had lower NSS at all time points post-WDI; the precise timing of when these differences became significant (*p* ≤ 0.05) was at the 24 h time point (Figure 2). Over time, *fat-1* mice demonstrated progressive improvements in overall neurological and behavioral motor deficits post-WDI. An ~88% improvement in the NSS task success rate by *fat-1* mice is observed comparing NSS at the 1 h vs. the 168 h time points (1.5 ± 0.3 and 0.2 ± 0.2, respectively). Although a similar trend was also observed in WT mice, there was only a 41.5% improvement in NSS comparing 1 h vs. 168 h (2.0 ± 1.0 and 1.2 ± 0.3, respectively). The average NSS remained elevated for the WT mice throughout the assessment period, relative to their *fat-1* littermates. Sham *fat-1* and WT mice had NSS scores of 0–0.5 in the first 24 h (data not shown).

### 3.3. Brain Fatty Acid Composition

Fatty acid compositional analysis of brain total lipids showed that *fat-1* mice expressed a phenotype with significantly (*p* ≤ 0.05) higher n-3 PUFA, primarily from DHA (18.04 ± 0.34), in contrast to their WT counterparts, that had significantly higher levels of n-6 PUFA from arachidonic acid, 20:4n6 (17.90 ± 3.16) (Table 1). 

### 3.4. Cerebral Microhemorrhage

**Figure 3:**
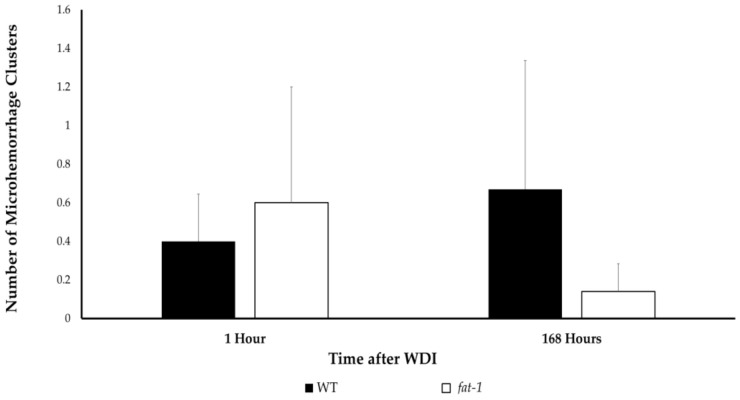
Number of microhemorrhage clusters for mice at 1 h (1 male and 4 female WT; 3 male and 2 female *fat-1*), and 168 h (4 male and 2 female WT; 3 male and 4 female *fat-1*), post-WDI. No significant difference (*p* > 0.05) was found at either time point by Student’s *t*-test. Bars represent mean ± SEM.

**Figure 4:**
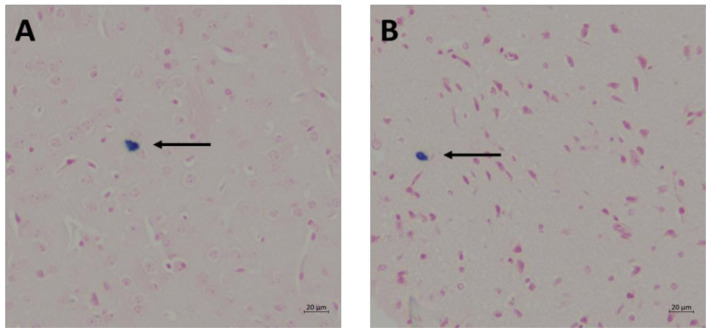
Images of sagittal brain sections of WT and *fat-1* mice stained with Prussian blue 1 h post-WDI. Images were taken with a Zeiss AxioZoom V.16 at 11.2× magnification. Arrows indicate positive Prussian blue granules (i.e., microhemorrhage). (**A**) WT; (**B**) *fat-1*.

There were no statistical differences (*p* > 0.05) in the average number of microhemorrhage clusters when comparing WT to *fat-1* mice in the 1 h and 168 h termination groups post-WDI (Figure 3 and Figure 4). 

## 4. Discussion

This study demonstrates that higher brain DHA was associated with the mitigation and rapid recovery of motor and behavioral deficits due to a WDI in the *fat-1* mouse model. The use of a genetic model demonstrates the cause and effect between the presence of DHA in the brain and the outcomes related to the WDI. 

### 4.1. Motor and Behavioral Function

Nonpathological indices used to verify mTBI in rodent models are frequently limited to brain imaging techniques (e.g., positron emission tomography, computed tomography, or magnetic resonance imaging), which are costly and often not readily available to most preclinical research laboratories [16]. This indicates a need for functional assays that are able to detect clinically relevant changes in behavior that correlate with underlying biological changes [16]. The NSS represents such an assay; this test is easy to administer, assesses numerous deficits (i.e., motor and behavioral), and it is associated with the degree of brain damage, as evidenced by in vivo MRI and histological mouse studies [17]. Therefore, unlike other functional assessments, which may only convey information pertaining to only one injury outcome (e.g., Rotarod, the wire grip test, etc.), the NSS test provides a simple, accessible, and reliable method for determining the overall neurological status of the animals. The NSS results of this study demonstrate that *fat-1* mice have consistently lower NSS at all time points post-WDI, relative to their WT counterparts. Since the NSS is a composite score, it can be assumed that improvements in either one, or a combination, of the aforementioned domains may account for the lower NSS identified in *fat-1* mice. 

It is possible that the elevated n-3 PUFA and lower n-6 PUFA in the *fat-1* mice conferred neuroprotective properties, thereby sparing some neurologic function, which allowed them to be more successful in completing the NSS tasks relative to their WT counterparts. Albeit, a slight distinction, it is also the lack of DHA in the brain that results in a poorer NSS. This is important because the supraphysiological levels of brain DHA are not apparent in the *fat-1* mouse [18]. While not measured in this study, n-3 PUFA has been shown to upregulate the brain-derived neurotrophic factor, involved in motor and brain function, and, thus, it is a potential mechanistic pathway for future investigation [19]. 

It is also possible that the ameliorations in the motor impairments were the impetus for the rapid decline in NSS observed in *fat-1* mice relative to their WT counterparts post-WDI. Stiffness in the lower extremities, instability, and coordination deficits, in conjunction with cognitive declines after a mild concussive injury in athletic populations, have also been documented [20,21,22,23,24]. While future studies should aim to elucidate the exact mechanisms underlying the potential role of n-3 PUFA in mitigating motor deficits, recent literature has proposed that n-3 PUFA has the capacity to improve information processing and the allocation of appropriate resources to match the demands of a task via improved cortical networking [23,24]. Further, the normalization of BDNF concentrations due to n-3 PUFA may confer protective properties on locomotor function by increasing synaptic facilitation and neuronal excitability, although this requires further study in this model [19].

It is also possible that improvements in behavioral function contributed to the lower NSS in *fat-1* mice across all time points. Behavioral disturbances have also been reported in subjects that have been affected by a concussive injury [7]. However, findings pertaining to the relationship between n-3 PUFA concentrations and behavior are scarce in the literature. Thus, additional investigation is necessary to elucidate the existence of the possible mechanistic role of n-3 PUFA in reinstating appropriate behavioral patterns following a concussion.

### 4.2. Time to First Movement 

We also used time to first movement to assess the protective properties of n-3 PUFA on neurological restoration. In a previous study by Kane et al., the righting reflex response was evaluated in 12 mice as an indicator of neurologic restoration [11]. This response was defined as the time taken by the injured mice to instinctively return to a prone position post-injury and/or anesthesia. Our study took a slightly modified approach and, instead, utilized the time to first movement post-WDI as an indicator of neurologic restoration. This was done to comply with concerns raised by the Animal Care and Use Committee that placing the mice on their backs post-WDI may cause undue stress, thus confounding the results.

The significantly faster times to first movement observed in *fat-1* compared to WT mice indicates that n-3 PUFA increases neurological restoration post-concussion. This can likely be attributed to the neuroprotective and neurorestorative properties conferred by n-3 PUFA [4].

### 4.3. Cerebral Microhemorrhage 

The immunohistochemical results of this study demonstrate that there are no significant differences in the prevalence of cerebral microhemorrhage when comparing *fat-1* to WT mice 1 h and 168 h post-WDI. This is in agreement with previous work suggesting that cerebral microhemorrhages are not a prominent characteristic of mTBI. For example, while traumatic microhemorrhages may be present in TBIs of all severities, a prospective, observational study, comprised of 439 subjects, reported that microhemorrhages were identified in merely 27% of patients with mTBI, 47% of patients with moderate TBI, and 58% of patients with severe TBI [25]. It is possible that exploring the utility of n-3 PUFA in protecting against microhemorrhages might be more relevant in the context of moderate to severe cases of TBI, rather than mTBI as investigated in this study. Instead, other assays that investigate the hallmark features of mTBI, such as inflammation, should be employed to elucidate the underlying biological changes that occur post-mTBI that are responsible for the functional deficits detected by the NSS.

### 4.4. Methodology to Study the Prophylactic Effect of n-3 PUFA in Concussion Prevention

The mTBI model reported in this study overcomes the limitations intrinsic to other animal models. Biomechanical analyses of head impacts sustained in athletic settings have shown that a high-velocity impact and rapid head acceleration are the most critical components in imparting a mild concussive brain injury [26,27,28,29,30,31,32,33]. However, this essential factor is not reflected in most existing animal models [32]. For instance, although mTBI models, such as the lateral fluid percussion and the controlled cortical impact, incorporate direct loading of the brain, these complex and costly methods fail to mimic the necessary swift changes in head acceleration [34,35]. In spite of their prevalent usage in the literature, the absence of the aforementioned features ultimately renders these mTBI models less ideal for reproducing the types of injury exhibited subsequent to a mTBI in humans.

Further, recent epidemiological studies have demonstrated that 85–89% of TBI patients acquired their injuries following a blunt closed-head trauma induced by traffic accidents and falls [36,37,38]. By contrast, penetrating injuries were defined as those penetrating the dura, and were reported in solely 0.8–3% of cases [36,37,38]. Hence, a mechanical, high-velocity impact on the skull causing rapid acceleration certainly imitates, e.g., a fall, a motor vehicle ejection, much more accurately than models that implement complex surgical procedures, or those wherein the subject’s head is fixed to a platform during impact [12]. 

The model described in this study allows for the delivery of a concussive impact to the closed cranium of an unrestrained subject, thus permitting for the swift acceleration of the head and torso. The confounding effects on the mTBI outcome that accompany prolonged exposure to anesthetic agents are also minimized in the current model, as only light anesthesia is necessary [39]. Owing to the fact that mice recover spontaneously, and surgical preparations are not required, this simplifies the procedure and renders its execution rapid and inexpensive. More importantly, because the described technique replicates the mechanistic features of a human mTBI, it induces clinically relevant behavioral outcomes that are representative of the constellation of symptoms that occur after a concussive injury—collectively denoted as “post-concussive syndrome” [2,3,9,11,29]. Ultimately, this model facilitates the general transferability from “bench to bedside”.

The use of the genetically engineered *fat-1* mouse in the present study offered a novel opportunity to investigate the prophylactic role of n-3 PUFA in mitigating the pathological cascade that follows a mTBI. Traditionally, the modification of the tissue nutrient composition in animal nutrition studies is achieved by dietary supplementation. Although it is a widely accepted practice to study the effects of nutrients on a vast number of physiological processes and pathologic situations by supplementing experimental groups with different diets, this approach introduces potentially confounding variability [40]. The major confounder is the isocaloric requirement in diet studies. Thus, it is not possible to differentiate whether an effect is attributed to the addition or removal of a given macronutrient. In studies examining the effects of n-3 and n-6 PUFA, experimental groups of animals are fed two different diets exhibiting differing n-3/n-6 PUFA ratios to establish different fatty acid profiles [40]. Typically, fish oils and plant seed/vegetables oils are utilized as n-3 and n-6 PUFA supplements, respectively [40]. Hence, because of the differences in the sources from which these PUFA are derived, they are likely to exhibit traces of other bioactive compounds that, despite their concentrations, are likely to influence the study outcomes [40]. Another valuable benefit that this approach presents is that transgenic *fat-1* mice have the ability to endogenously convert n-6 to n-3 PUFA as early as in the embryo stage until end-of-life [40]. This removes the lengthy and expensive feeding period that is often necessary to modify the tissue nutrient profile in conventional dietary supplementation studies. The *fat-1* mouse model served as an ideal approach to eliminating the need for n-3 PUFA supplementation, providing a genetic approach for studying the prophylactic role of n-3 PUFA. Thus, mechanistically, the changes in n-3 PUFA result in complementary changes in n-6 PUFA, reflected in the higher brain DHA in the *fat-1* mice, and lower n-6 PUFA, including arachidonic acid (20:4n-6) (Table 1). While both n-3 and n-6 PUFA serve distinct biological roles in the brain, from a causal pathway perspective, increased DHA is the initial driver of such changes in the *fat-1* model. Similarly, in support of a causal role of DHA, mice receiving a cortical impact injury and treated post-injury with DHA via i.v. injection were also shown to have reduced neurological impairments [41]. Together, these observations support a role for DHA as a driver leading to the mitigation of brain injury outcomes. 

### 4.5. Limitations and Future Directions

This pilot study established a robust model and phenotype and showed promising benefits of n-3 PUFA for mitigating mTBI outcomes, and studies of larger sample sizes with a mechanistic focus are warranted. Male and female mice were pooled in this small study, but further exploration of potential sex-specific effects are warranted, given that protective effects were observed in both sexes. Effective concussion interventions should also be investigated targeting all aspects of the secondary injury while simultaneously initiating the repair, regeneration, and protection of the brain tissue. Such treatments should address the symptoms that persist long after the infliction of the primary injury (post-concussive symptoms, e.g., headaches, auditory disturbances, depression, memory deficits) [2,3,9].

## 5. Conclusions

In summary, the results from this pilot study suggest that n-3 PUFA has the potential to mitigate the motor and behavioral impairments arising from a mTBI. However, further research is required to determine the molecular mechanisms of action, the chronic effects of n-3 PUFA on long-term outcomes as well as the potential for n-3 PUFA to mitigate the effects of repetitive concussive injuries on overall brain health and function. 

## Figures and Tables

**Table 1:** Percent fatty acid composition of mouse brains.

Fatty Acid	WT	*fat-1*
16:0	28.25 ± 1.07	24.34 ± 0.31 *
18:0	28.29 ± 1.02	25.79 ± 0.25
18:1c9	17.56 ± 0.71	19.13 ± 0.61
18:2n6	1.28 ± 0.01	1.41 ± 0.15
20:3n6	0.33 ± 0.01	0.46 ± 0.03 *
20:4n6	17.90 ± 3.16	11.03 ± 0.03 *
20:5n3	0.15 ± 0.02	0.26 ± 0.02 *
22:4n6	3.52 ± 0.07	2.50 ±0.16 *
22:5n6	10.27 ± 0.91	0.43 ± 0.11 *
22:5n3	0.22 ± 0.02	0.27 ± 0.05
22:6n3	6.56 ± 0.98	18.04 ± 0.34 *

Values are mean ± SEM. * denotes significant difference (*p* ≤ 0.05) by Student’s *t*-test. Fatty acid analyses were assessed from WT (3 female) and *fat-1* (3 female) mice.
